# Buprenorphine Induction in a Rural Maryland Detention Center During COVID-19: Implementation and Preliminary Outcomes of a Novel Telemedicine Treatment Program for Incarcerated Individuals With Opioid Use Disorder

**DOI:** 10.3389/fpsyt.2021.703685

**Published:** 2021-10-28

**Authors:** Annabelle M. Belcher, Kelly Coble, Thomas O. Cole, Christopher J. Welsh, Anna Whitney, Eric Weintraub

**Affiliations:** Division of Addiction Research and Treatment, Department of Psychiatry, University of Maryland School of Medicine, Baltimore, MD, United States

**Keywords:** correctional settings, medications for opioid use disorder (MOUD), jail, carceral treatment, medications for addiction treatment (MAT), buprenorphine, opioid agonist therapy (OAT), telemedicine

## Abstract

Over 10 million individuals pass through U.S. detention centers on an annual basis, with nearly two-thirds meeting criteria for drug dependence/abuse. Despite proven efficacy, treatment with medications for opioid use disorder (MOUD) is underutilized in jail settings—a gap that could be addressed using telemedicine. Here we describe a new program of telemedicine-based clinical provision of new/continuing buprenorphine treatment for individuals detained in a rural jail. Implementation objectives were completed between January and August 2020, and patient encounters were conducted between August 2020 and February 2021. We established (i) telemedicine hardware/software capability; (ii) a screening process; (iii) buprenorphine administration methods; (iv) necessary medical release procedures; (v) telemedicine encounter coordination and medication prescription procedures; and (vi) a research platform. Seven incarcerated patients have been treated, two of whom were referred from community treatment. Patients were mostly male (71%), non-Hispanic White (86%), and averaged 33 years old. All patients tested positive for an opioid upon intake and began/continued buprenorphine treatment in the jail. Average time to first MOUD appointment was 9 days and patients were maintained in treatment an average 21 days. Referrals for continuing community treatment were offered to all patients prior to discharge. We report successful implementation of telemedicine MOUD in a rural detention center, with treatment engagement and initiation occurring prior to the high-risk period of discharge. The fact that this program was launched during the height of the pandemic highlights the flexibility of telemedicine-based buprenorphine treatment. Challenges and obstacles to implementation of buprenorphine treatment in a correctional system are discussed.

## Introduction

The United States is entrenched in an opioid epidemic that has disproportionally impacted rural areas of the United States (1, 2). Health care challenges that are endemic to non-metropolitan areas, such as geographic constraints, resource limitations and limited availability of specialty treatments, have been exacerbated by negative perceptions of medications for opioid use disorder (MOUD). Resultingly, the rural opioid problem is greater in scope and scale than that of urban areas (3, 4). Methadone, buprenorphine, and long-acting naltrexone are FDA-approved frontline treatments in community and hospital settings for opioid use disorder (OUD); but in rural areas of the U.S., their availability and uptake is limited (5, 6).

This lack of access poses a particularly acute problem for treatment within the criminal justice system. More than 10 million individuals pass through U.S. detention centers on an annual basis, and it has been estimated that as much as two-thirds of this population meet criteria for drug dependence or abuse (7)—a gross over-representation of the incidence rate observed in general (non-incarcerated) populations. With conservative estimates that up to 36% of all individuals with an opioid problem pass through U.S. corrections systems each year (8), OUD is highly prevalent in justice-involved populations. An estimated 15% of incarcerated individuals have an OUD (9, 10). Overdose risks associated with transition from prison to the community is particularly high in the 2 weeks following release and has been shown to be the leading cause of death for recently discharged individuals (11–13). Randomized controlled studies have shown that prison-initiated MOUD treatment greatly improves post-release outcomes on a host of measures, including retention in treatment, social function, and recidivism (14). In the United States, jails and detention centers serve as temporary confinement spaces for individuals who commit minor offenses, or who are awaiting trial for more serious offenses. Jail and detention center settings not only oversee individuals struggling with substance use disorders and withdrawal but are also in a unique position to initiate treatment in a controlled, safe environment. Unfortunately, criminal justice detainees have the least access to MOUD treatment (15)—particularly in rural areas (16).

Telemedicine provides a viable solution for health care and treatment gaps. Capitalizing on technological advances and secure Health Insurance Portability and Accountability Act (HIPAA)-compliant videoconferencing, telemedicine involves remote therapeutic encounters with “doctors on the screen” who interact directly with their patients to provide assessments, psychiatric care, and medication prescription. This method of healthcare provision has broken the significant barrier of geographic distance to promote equitable access to healthcare (17). Although reports of its use in carceral settings is limited, the available literature suggests that as a cost-effective and acceptable method of mental health service provision, telemedicine should be more widely adopted as a tool to increase healthcare access for confined populations (18). More recently, its utility has been underscored by the COVID-19 pandemic. No longer simply a solution to rural health care access, telemedicine has taken center stage as a major health care delivery platform during the COVID-19 public health emergency (19, 20).

Our Division of Addiction Research and Treatment within the Department of Psychiatry at the University of Maryland School of Medicine has been providing MOUD *via* telemedicine to a variety of substance use disorder treatment programs throughout the state of Maryland since 2015. With partnership programs across the state, the presence and variety of telemedicine models we provide is strongest in underserved rural counties in Maryland (21, 22). The overlap of OUD and criminal justice involvement drove our team to look for ways in which to access vulnerable jail populations to provide OUD treatment prior to the risky period of discharge. In the United States, methadone can only be dispensed through federally regulated Opioid Treatment Programs and is not a practical option in rural county jails; thus, our program focuses on providing treatment with buprenorphine.

Here we describe implementation and pilot evaluation of a novel jail telemedicine program to provide buprenorphine treatment to individuals who are incarcerated in rural Maryland detention centers. In the United States, jails and detention centers serve to temporarily confine pre-trial suspected offenders or individuals accused of minor crimes. Detention centers and jails usually do not keep individuals for periods more than 18 months. Considering differences in individuals' length of stay and readiness to accept treatment, a goal of our program is to initiate treatment as close as possible to an individual's intake into the jail to maximize the impact of our telemedicine-based buprenorphine clinical intervention as well as the potential for early and sustained engagement prior to release. Program implementation began in January 2020, the same month as Federal and local declarations of an infectious disease outbreak and public health emergency. We provide a description of the implementation process and integration into standing jail procedures, *de novo* build-out of the hard and software for the telemedicine platform and data collection, and procedures for screening and referral for treatment. Further, we report pilot results of the initial cohort of telemedicine-based buprenorphine treatment initiates, describe patient demographic and drug use history characteristics, and report on buprenorphine treatment within the jail setting. Finally, we describe our experience with the challenges and barriers to telemedicine-based buprenorphine implementation in a rural jail.

## Materials and Methods

Results are reported following the Standards for Quality Improvement Reporting Excellence (SQUIRE) guidelines (23).

### Setting

With a population of ~38,000, Talbot County is classified by the Federal Office of Rural Health Policy as a rural area. The Talbot County Detention Center is a 148-person-rated facility with an average daily pre-COVID census of ~60–80 individuals and serves as the designated central booking center for the county. The average length of stay is 6 months, but individuals awaiting trial can be held for up to 18 months. Prior to our program, the only individuals who were offered MOUD within the jail were pregnant women who were already engaged in treatment within the community; otherwise, supervised withdrawal management with medications for symptom relief (e.g., loperamide, acetaminophen, meclizine) was the standard operating procedure for individuals presenting with an opioid use disorder at jail intake. Data reported were obtained on incarcerated individuals enrolled into the telemedicine-based buprenorphine program from August 15, 2020 to February 15, 2021.

### Project Development Activities

#### Meetings With Detention Center and Health Department Staff

**Figure 1:**
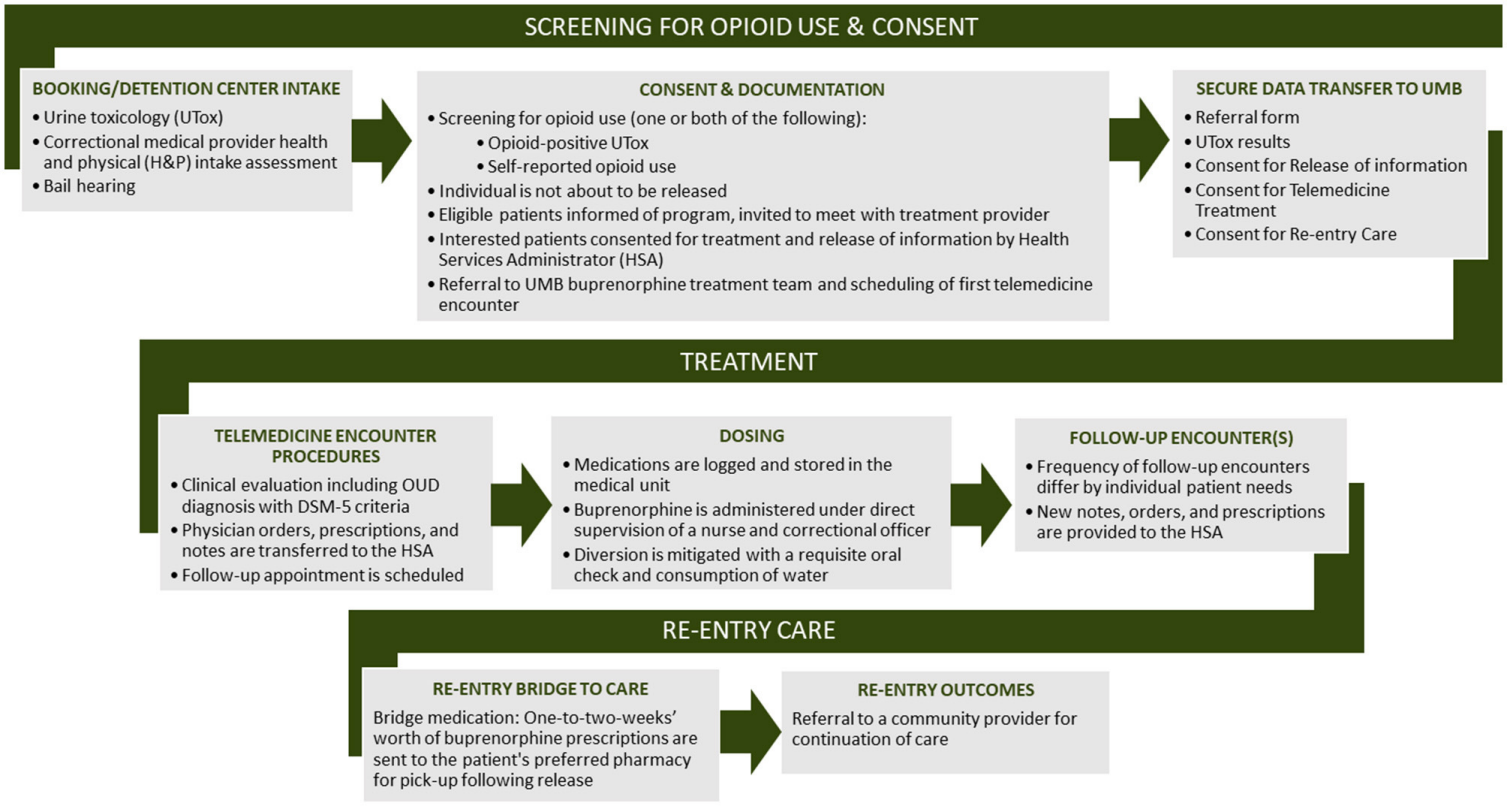
Flow diagram depicting coordination and provision of clinical telemedicine-based buprenorphine care.

In order to ensure seamless contact with key individuals involved in the program and to address concerns prior to telemedicine-based buprenorphine implementation, regular meetings were established that included the Talbot County Detention Center leadership and correctional staff, the health officer and local addictions authority, and contracted jail health care providers (WellPath nursing staff and medical directors). The first several of these meetings were held in person, but subsequent to the onset of the pandemic, have been held as bi-monthly or monthly virtual (Zoom) teleconference meetings. These meetings allowed us to identify a point-person for the delivery of medications, release of healthcare information from the jail records (urine toxicology, withdrawal assessment, etc.) to guide buprenorphine best clinical practices, and coordination of the telemedicine encounters. Through an iterative process, development of a protocol for telemedicine-based buprenorphine clinical care delivery was established, a flow chart for which is depicted in Figure 1.

#### Medications Procurement, Storage, and Dispensation

Buprenorphine mono-product (Subutex) is provided as either 2 or 8 mg tablets once daily. Medications are administered either by the health services administrator (HSA) or other certified personnel. Starting dose blister packs are stored in the facility's double-locked medical cart to which only the jail's authorized medical staff members have access. Following an initial medical evaluation, patient-specific buprenorphine prescriptions are ordered from the correctional pharmacy service provider (CorrectRx) and delivered weekly. All medication administration is recorded in a controlled substances log.

#### HIPAA-Compliant Communication and Transfer of Electronic Health Information

Clinical encounters are logged into an electronic health record database (Epic [Epic Systems Corporation]), and documentation is maintained at the University of Maryland. Microsoft Teams, a HIPAA-compliant platform that is housed and maintained within the University of Maryland School of Medicine and protected by a firewall to ensure secure transfer of sensitive information, is used to enable confidential exchange of health information from the jail healthcare staff to the treatment team.

#### Drafting and Finalization of Requisite Telemedicine-Based Buprenorphine Treatment Forms

In order to initiate telemedicine-based buprenorphine services at the detention center, incarcerated individuals must consent to a release of the health and physical form (H&P) that is given to all new jail intakes. Several forms necessary for telemedicine-based buprenorphine treatment were developed; these included Release of Information, consent to telemedicine treatment, consent to treatment with buprenorphine, H&P data extraction (filled by a member of the jail's healthcare nursing provider team upon intake into the jail), buprenorphine prescription pads, and a medical progress note.

### Teleconferencing Equipment

All interactive video conferencing sessions are conducted either point-to-point or multipoint using an Advanced Encryption Standard (AES) algorithm via Internet Protocol (IP) connections. Bridging of calls occur either through the multipoint software license of the video conferencing system or using the UMB-sponsored account for Cisco WebEx or Zoom. The UMB-sponsored account has the required security compliance documents that ensure high levels of security for confidential content covered under state and federal laws and regulations (e.g., HIPAA).

Polycom video conferencing devices are supported by internal IT staff and secured by Cisco Video Expressways. All video sessions are secured by AES-256-bit encryption, and all equipment and software used for telemedicine encounters has been deemed HIPAA-compliant by the University of Maryland School of Medicine Information Security Office. All video calls are logged on the Polycom endpoint and the Cisco expressways.

COVID-19 restrictions precluded us from entering the jail and establishing a full DX-80 installation, the standard telemedicine hardware utilized by our telemedicine-based buprenorphine clinical service programs. To circumvent this barrier, we purchased a small, inexpensive laptop on which to hold telemedicine encounters; all encounters described in this report were conducted using this temporary telemedicine infrastructure.

### Brief Screening and Referral for Treatment

Briefly, all clinical services that are conducted in-person (consent for treatment, brief screening, referral for treatment, and urine toxicology) are conducted by the HSA, whereas all substance use disorder clinical encounter procedures (diagnosis and prescribing) are conducted remotely (via telemedicine) by physicians based at the University of Maryland. As part of standard intake procedures, individuals who are newly booked into the detention center provide a urine sample and a self-report of lifetime drug use to staff at the detention center, either at the time of correctional intake or at the time of telemedicine-based buprenorphine referral. Individuals with active opioid use prior to arrest are placed on a withdrawal management protocol in which the Clinical Opioid Withdrawal Scale (COWS) is administered by the HSA. The HSA also provides supportive medication treatment for symptoms associated with opioid withdrawal, including non-steroidal anti-inflammatory drugs and anti-emetics. In addition to this palliative acute care by the HSA, treatment for more severe symptoms would be addressable by the detention center's intensive outpatient treatment psychiatrist, who was available in case of emergencies. Individuals who screen positive for OUD, are not released on pre-trial bail, and are interested in hearing about telemedicine-based buprenorphine, are referred by the HSA or behavioral health coordinator (BHC) to the telemedicine-based buprenorphine provider affiliated with the University of Maryland. At this time the HSA/BHC obtains a telemedicine consent and a release of information (ROI) consent. The HSA/BHC then transfers the referral form, urine toxicology results, Clinical Opioid Withdrawal Scale (COWS) assessment results, nursing notes, and the above consent forms to the provider via Microsoft Teams (a secure cloud-based platform). Once received by the provider, the individual is added to a provider's schedule and a medical health record is created at the distant site. Providers review the above documents prior to the telemedicine-based buprenorphine encounter. After the telemedicine-based buprenorphine encounter, and if the provider determines that the individual meets the medical criteria for MOUD, a treatment consent is obtained by the HSA/BHC prior to dosing.

### Buprenorphine Induction and Maintenance

Initial buprenorphine dosing is patient-centered and is decided by the individual practitioner based on a thorough history and clinical exam. Given that new patients tend to be opioid-free for more than a week, a starting dose of 4 or 8 mg is used. Both a prescription and administration order for the medication are transmitted electronically through a secure platform. Patients are administered buprenorphine daily between the hours of 8:00 a.m. and 9:00 a.m. within the secured medical unit under directly observed therapy conditions. The HSA/dosing nurse and a correctional officer are both present during dosing procedures. Prior to consuming the buprenorphine medication, patients are instructed to consume a cup of water. The buprenorphine is crushed by the nurse and then placed under the patient's tongue. The patients are instructed to sit on their hands and are monitored by a correctional officer. After the medication is fully dissolved, a subsequent cup of water is consumed. Prior to leaving the medical unit, patients are given a_visual mouth inspection to ensure complete consumption of the_medication._Additionally, patients' detention center provided clothing pockets are inspected to ensure diversion does not occur. Jail medical staff are provided physicians' after-hours contact information to facilitate ongoing communication at any point during treatment.

### Data Collected

Data are collected from detention center health records using the data extraction sheet described above, and from electronic health records logged in Epic. De-identified data are stored on a database created in REDCap, a HIPAA-compliant database for real-time data entry and validation, storage and retrieval (24). Routine data backups are conducted by the Department of Psychiatry Information Technology group. All data are collected as part of a study protocol approved by the University of Maryland's Human Research Protection Office (UMB IRB protocol No. HP-00090980). Data collected and reported include patient demographic characteristics (sex, age, race, ethnicity, other mental health diagnosis, family history of either substance use or mental health disorder and marital status), drug use characteristics [self-reported number of years of opioid use, frequency of use, most recent route of administration, intake toxicology results, withdrawal score as assessed by the COWS (21)], criminal justice data (reason for conviction and whether or not convicted), and telemedicine-based buprenorphine treatment data pre- and post-discharge (transferred from community buprenorphine treatment, buprenorphine doses across treatment, number of days between correctional intake and first telemedicine-based buprenorphine appointment, total number of follow-up in-custody appointments, total number of days in telemedicine-based buprenorphine care, and referral to post-discharge continuing care and type of referral).

### Confidentiality

Personal health information, including urine drug screen results, is only accessible by staff providing direct medical care to patients. Further, personal health information is only shared with the necessary detention center, health department, and University of Maryland staff for the purposes of care coordination. This information is protected under the federal regulations governing Confidentiality and Drug Abuse Patient Records, 42 CFR, Part 2, and the Health Insurance Portability and Accountability Act of 1996 ('HIPAA'), 45 CFR. pts 160 & 164. Disclosure of any PHI is provided via written consent for a period of 1 year, per the ROI signed by the patient at the time of telemedicine-based buprenorphine referral. All equipment was registered to the Psychiatry video expressways for HIPPA compliant 128-bit AES security and easier dialing.

### Urine Drug Screens

All individuals are tested at one time for drugs (and if female, for pregnancy) on the day of intake into the jail. Analytes measured provide screening for recent use of the following substances: oxycodone, morphine, fentanyl, propoxyphene, amphetamine, methamphetamine, cocaine, 5-methylenedioxymethamphetamine [MDMA], benzodiazepine, methadone, cannabis, barbiturates, PCP, tricyclic antidepressants, and buprenorphine.

### Statistical Analysis

Frequencies and proportions are reported for discrete variables and means and standard deviations are reported for continuous data. Statistical analyses were conducted using SPSS v.26.

## Results

### Patient Baseline Characteristics

A total of seven incarcerated patients with OUD were offered the opportunity to enroll into the telemedicine-based buprenorphine program from December 15, 2020 to February 15, 2021, and all seven patients accepted and consented to treatment. Baseline characteristics are described in Table 1; briefly, the treated population was majority male (71%) and White/Caucasian (86%) with an average age of 33 years old. Reasons for incarceration varied, but the most common booking charge was violation of probation (43%). Average length of stay was 33 days (range = 21–71 days). All subjects self-reported a history of opioid use, with an average of 8.4 years of use. Insufflation was the most commonly reported route of administration (43%).

**Table 1:** Characteristics of incarcerated patients (*N* = 7, unless otherwise noted).

**Patient characteristics**	***n* (%)^a^**
Age [mean (SD)]	33.4 (8.3)
**Sex**	
Male	5 (71)
Female	2 (29)
**Race**	
White/Caucasian	6 (86)
Black/African-American	1 (14)
Hispanic/Latin-X Ethnicity	0
Married or significant other (*n =* 6)	2 (29)
**Self-reported co-morbid mental health condition (*****n** **=*** **6)**^b^	
Depression	3 (43)
Anxiety	2 (29)
Other^c^	3 (43)
No other co-occurring	3 (43)
Family history of substance Use (*n =* 6)	4 (57)
**Reasons for incarceration**	
Assault	2 (29)
Probation violation	3 (43)
DUI/DWI	1 (14)
Driving on suspended license	1 (14)
Convicted of charge	4 (57)
Length of Stay [Mean (SD)]	33 (18)
Years of opioid use [Mean (SD); *n =* 6]	8.4 (3.7)
**Route of opioid administration (*****n** **=*** **6)**	
Insufflation (Intranasal; IN)	3 (43)
Intravenous (IV)	2 (29)
IN and IV	1 (14)
**Urine toxicology positive screening**	
Opioids^d^	4 (57)
Psychostimulants^e^	2 (29)
THC	3 (43)
Tricyclic Antidepressants	1 (14)
Methadone^f^	3 (43)
Buprenorphine^g^	2 (29)
Days incarcerated prior to first tMOUD encounter [Mean (SD)]	9 (11)
**Buprenorphine dose [Median (Range)]**	
Induction	8 mg (4–20 mg)
One-week^h^	12 mg (8–16 mg)
Final^i^	16 mg (8–24 mg)
Number of days in tMOUD treatment [Mean (SD)]	21 (9.5)
**Discharge outcomes**	
Linkage to treatment in the community	3 (43)
Transferred to higher level of care	2 (28.5)
Lost to follow-up	2 (28.5)

a *Percentages reported on a total n of 7; percentages not adding to 100% represent missing data*.

b *Not mutually exclusive*.

c *Other co-occurring diagnoses include bipolar (n = 1), obsessive compulsive (n = 1), panic disorder (n = 1), and ADHD (n = 1)*.

d *Positive screens included fentanyl (n = 4) and oxycodone (n = 1)*.

e *Positive screens included amphetamine (n = 1), cocaine (n = 1), and methamphetamine (n = 1)*.

f *Two patients were verified to have received prescribed methadone from a hospital or other area jail prior to intake*.

g *Both patients transferred into care from community buprenorphine treatment programs*.

h *One week discharge dose data is not provided for one patient (patient 006 voluntarily withdrew from treatment prior to discharge)*.

i *Final discharge dose data are not provided for two patients due (patient 004 requested a buprenorphine taper prior to discharge, and patient 006 voluntarily withdrew from treatment prior to discharge; see Discussion for further details)*.

### Urine Toxicology Screening Results

All patients tested positive for at least one substance on the urine drug screen panel, with fentanyl being the most common substance for which patients tested positive (57%). Other positives screens included oxycodone (*n* = 1), amphetamine (*n* = 1), cocaine (*n* = 1), tetrahydrocannabinol (THC, *n* = 3) tricyclic antidepressants (*n* = 1), methadone (*n* = 3), and buprenorphine (*n* = 2).

### Buprenorphine Treatment Within the Detention Center

The average number of days that lapsed between jail intake and the initial telemedicine-based buprenorphine encounter with our treatment team was 9 (range = 2–34). All seven patients were prescribed buprenorphine in the jail setting; five patients began a new treatment course, and two patients transferred from buprenorphine treatment in the community to continue treatment within the detention center. The median prescribed dose of buprenorphine on day 1 (induction) was 8 mg. Initial doses of buprenorphine for the five new treatment initiates were 4 mg (*n* = 1) and 8 mg (*n* = 4), and 16 and 20 mg for the two patients transferring from community treatment. The median 1-week post-initiation dose was 12 mg (*N* = 6; range = 8–16). Some patients (*n* = 2) discontinued buprenorphine treatment while incarcerated. Thus, the median final dose (prior to treatment discontinuation or discharge) for the 5 patients was 16 mg (range = 8–24). Except for one patient who had an existing buprenorphine prescription prior to incarceration and refused treatment after the third day of telemedicine-based buprenorphine treatment (patient 006), all patients were retained in treatment within the jail for at least 2 weeks prior to discharge. It is noteworthy that this same patient (006) who refused buprenorphine treatment also refused food or any type of jail-based treatment that was afforded (which included intensive outpatient psychiatric treatment). All patients remained in treatment for an average of 21 days (range = 3–35) and had a median number of 3 telemedicine-based buprenorphine encounters (range = 1–4). One patient (patient 004) had an unscheduled bail review ~2 weeks after beginning treatment. Due to COVID, the court allowed this patient to be released to an inpatient treatment facility, but the facility's house rules did not accept patients who were receiving methadone or buprenorphine. Further, the facility allowed this patient to receive a naltrexone injection prior to his release from jail; thus, this patient was tapered off buprenorphine for 7 days and was prescribed a single dose of naltrexone 1 week later.

### Treatment Upon Discharge

Treatment upon discharge outcomes varied for patients, and were dependent on several factors, including patient willingness to continue treatment in the community and court-ordered mandates surrounding release. Of the seven patients enrolled in the telemedicine-based buprenorphine program, three scheduled an appointment linking them to treatment in the community. These referred patients were provided bridge prescriptions to their preferred pharmacy location to enable continuation of medication in the community following discharge. Two patients were transferred to a higher level of care (inpatient treatment for their substance use disorder), and two patients were discharged and lost to follow-up. Of the two patients with existing buprenorphine prescriptions, one refused treatment while incarcerated and one scheduled an appointment at their previous treatment center located within the community.

## Discussion

The extremely high morbidity and mortality associated with overdose upon discharge from incarceration has been underscored in multiple reports across a variety of carceral settings and geographic locations (11–13, 25–29). With meta-analysis findings that the first 2 weeks of discharge carry a three- to 8-fold increased risk of drug-related death (11), it is not possible to overstate the urgent need for interventions that reach individuals prior to their release from jails and prisons. The positive data showing improved outcomes when pharmacotherapies are introduced is unwavering (30): MOUD provision from within correctional facilities prevents overdose upon release (31). Recognizing the need for treatment services for this vulnerable sub-population, in 2019 the state of Maryland passed legislation mandating that all state and local correctional facilities make all three FDA-approved medications for opioid use disorder (methadone, buprenorphine and naltrexone) available by 2023 (HB-116) (32). Although these moves to increase MOUD institutional access are encouraging, this standard of care for OUD is minimally available in Maryland jails, and even less so for jails in vulnerable rural areas (16). Telemedicine offers a viable solution for rural jail MOUD access across the U.S.—particularly in light of the global pandemic.

Our group has initiated several novel telemedicine-based buprenorphine programs since 2015, partnering with addiction behavioral treatment facilities and health departments in rural areas throughout the state of Maryland (22, 33, 34). The goal of these various programs is to fill an important addiction treatment gap in areas hard-hit by the opioid epidemic. At each of these remote sites, the program virtually connects OUD-diagnosed individuals with addiction medicine-trained physicians capable of providing comprehensive evaluations and treatment with buprenorphine. The outcomes from these various telemedicine-based buprenorphine programs are extremely encouraging and demonstrate a clinical benefit, with results comparable to those reported by direct (face-to-face) treatment. Chart reviews of buprenorphine-treated patients enrolled in our telemedicine-based buprenorphine programs demonstrate a 50–60% 3-month retention rate, with only 6–7 of those individuals testing positive for continues opioid use (22, 33, 34). The current initiative to provide treatment from within a detention center located in one of the areas where we have traction represents an important outgrowth of existing programs to reach at-risk incarcerated patients before they are released into the community. Our primary goal in instituting this program is to ensure the availability of medications for OUD pre-release to mitigate overdose risk upon discharge. However, treatment in jail offers several important patient-centered benefits, including the prevention of withdrawal and patient stabilization—factors that increase the individual's chances of beginning the recovery process to benefit from the non-medication treatments that are offered by the jail.

We were able to successfully implement a novel clinical MOUD program in a rural jail that, prior to our program's initiation, was not able to offer medications for OUD to the general census of incarcerated individuals. We established the initial groundwork and logistics and have created a mechanism to offer treatment to newly incarcerated individuals who need it. Initial outcomes from this initiative are favorable, with a 100% acceptance rate: all seven individuals who were offered treatment chose to receive it. With the exception of one patient who refused treatment after 3 days of buprenorphine treatment, the majority of individuals were maintained within our care while incarcerated and were either retained in buprenorphine treatment for the duration of their incarceration (*n* = 5) or were tapered and transitioned to naltrexone at the patient's request (*n* = 1).

### COVID-19 Considerations

Slated to begin in the first quarter of 2020, implementation of our telemedicine-based buprenorphine in jail program co-occurred with the emergence of the COVID-19 public health emergency. In response to the pandemic, federal regulations surrounding OUD treatment were eased to allow for the use of telehealth-based platforms (both video and phone) for clinical encounters. These included changes to Medicare/Medicaid allowances, loosened requirements for in-person initiation of buprenorphine, and the easing of restrictions of non-HIPAA-compliant communication platforms (35). The pandemic precluded several planned activities of the program, including in-person visits to the jail, the hiring of on-site staff, and the installation of standard DX-80 telemedicine equipment. Despite these forced alterations, our team was able to implement nimble solutions: meetings with jail and medical staff were held *via* Zoom, responsibilities were distributed among existing team members, and temporary “plug-in and go” telemedicine infrastructure was delivered to the jail. Although we will resume the full range of planned program development once the crisis has passed, it is unclear whether and to what degree the standing emergency OUD telemedicine regulations on the prescribing of controlled substances will return to pre-COVID standards. We and others have argued for the continuation of the relaxed federal policies surrounding MOUD treatment (36–38). Although COVID-19 vaccines are now publicly available as a solution for the global pandemic, the possibility exists that it may take months to years to achieve the vaccination coverage necessary for everyone to be protected (39). Thus, MOUD treatment that employs infection risk mitigation strategies, which include the provision of telemedicine, are indispensable for the foreseeable future.

Throughout the pandemic, efforts have been made at the county and state level to keep individuals out of high-risk environments such as jails and prisons. Thus, the local court system reduced restrictions on criteria for bail, and significantly decreased the number of individuals housed in the detention center. This had a direct impact on our ability to engage and recruit larger numbers of patients into the telemedicine-based buprenorphine program.

### Challenges

Beyond those incurred by COVID-19, our team experienced several challenges in the initiation of a telemedicine-based buprenorphine program in a rural Maryland jail. One challenge was in the appreciation of the multiple hierarchical authorities governing jail programs (Detention Center Directorship, Medical Staff Directorship, and local Health Department leadership) as well as the required logistics of obtaining approval for implementation (for example, a requisite memorandum of understanding with pass-through authorizations). Although our team achieved a nuanced understanding throughout the process of implementing our program, information regarding the necessary authorizations and requirements would have streamlined program development.

An ongoing challenge surrounds the unpredictability of individuals' entry and length of stay in the detention center. Our team benefited from a close collaboration with the intake medical staff to anticipate new intakes into the jail. Frequently, however, the outcomes of scheduled bail hearings would change determinations of a given patient's length of stay, which would have major implications for ongoing treatment. Early on, our team realized it was important to receive information from the patients themselves regarding their schedules to appear before the court. As an example of how this uncertainty impacted treatment in the detention center (also mentioned above), one individual initiated buprenorphine treatment while incarcerated. After having a bail-review hearing, this patient decided to take the judge's offer to enter an inpatient facility in lieu of incarceration. Unfortunately, however, this inpatient facility did not accept individuals who were prescribed buprenorphine. With only 1 week until release, the inpatient house rule prohibiting opioid agonist treatment forced the patient to titrate off buprenorphine rather quickly, and to receive a naltrexone injection at the time of release from the detention center—an unfortunate situation that could have been avoided with knowledge of the patient's impending early release.

Another challenge was in the shortage of staffing within the detention center. This limited the capacity of medical staff to provide MOUD care and consequently, limited the number of patients who were able to be referred to telemedicine-based buprenorphine treatment. Additionally, a lack of providers within the community limited the availability of provider options for those released after initiating MOUD care within the detention center. Lastly, providing MOUD care was new to the detention center, requiring the development and refinement of referral, screening, and post release care coordination processes.

## Limitations

One major limitation to this study is the small sample size, limiting the generalizability of our findings. Additionally, we anticipate that implementation will be different from one detention center to another; thus, methods described here that have worked at the Talbot County Detention Center may not be applicable at other sites.

## Conclusions

The ongoing opioid crisis continues to increase demand for addiction medicine provision, particularly in rural areas. Jails represent a unique access point to engage patients, but access to experienced addiction medicine providers is limited. Telemedicine closes this gap. Our successful pilot implementation of jail-based telemedicine-based buprenorphine treatment, with engagement and initiation occurring proximal to jail intake, is an encouraging demonstration of feasibility. The fact that this program was launched during the height of the pandemic highlights the flexibility and sustainability of telemedicine-based buprenorphine.

## Data Availability Statement

The raw data supporting the conclusions of this article will be made available by the authors, without undue reservation.

## Ethics Statement

The studies involving human participants were reviewed and approved by University of Maryland, Baltimore Human Research Protections Office. Written informed consent for participation was not required for this study in accordance with the national legislation and the institutional requirements.

## Author Contributions

AB: conceptualization, writing—original draft, funding acquisition (FORE), investigation (Chart Review), and formal analysis. EW: conceptualization, methodology, investigation (Patient Encounters), writing—reviewing and editing, funding acquisition (FORE), and supervision. KC: funding acquisition (MD Dept. Health), project administration, supervision, and writing—reviewing and editing. TC: data curation, project administration, investigation (Chart Review), visualization, and writing—reviewing and editing. CW: methodology, investigation (Patient Encounters), and writing—reviewing and editing. AW: visualization and writing—reviewing and editing. All authors contributed to the article and approved the submitted version.

## Funding

This work was supported by the Foundation for Opioid Response Efforts (FORE; EW, AB). Medication was subsidized with funds from the Talbot County Health Department (Maryland Department of Health, Talbot Local Addictions Authority). These funding sources had no role in the design of this study and did not have any role during its execution, analyses, interpretation of the data, or decision to submit results.

## Conflict of Interest

The authors declare that the research was conducted in the absence of any commercial or financial relationships that could be construed as a potential conflict of interest.

## Publisher's Note

All claims expressed in this article are solely those of the authors and do not necessarily represent those of their affiliated organizations, or those of the publisher, the editors and the reviewers. Any product that may be evaluated in this article, or claim that may be made by its manufacturer, is not guaranteed or endorsed by the publisher.
